# Continuous quality improvement in a community-wide TB screening and prevention programme in Papua New Guinea

**DOI:** 10.5588/pha.24.0013

**Published:** 2024-09-01

**Authors:** N.P. Pank, A. Aung, G. Kama, A. Murray, K.L. Huang, J. Greig, M. Bauri, G. Chan, C. Masah, K. Kaison, S. Umali, T. Peter, C. Wera, C. Velaki, M. Ase, I. Krangaie, R. Viru, T. Kurumop, T. Keam, S. Islam, W. Pomat, A. Maha, M. Boga, M. Kal, N. Wuatai, S.M. Graham, S.S. Majumdar, T. Marukutira

**Affiliations:** ^1^ Burnet Institute, Daru, Western Province, Papua New Guinea and Melbourne, Australia;; ^2^ Western Provincial Health Authority, Daru, Western Province, Papua New Guinea;; ^3^ PNG Institute of Medical Research, Goroka, Papua New Guinea;; ^4^ National Department of Health, Port Moresby, Papua New Guinea;; ^5^ Department of Paediatrics, University of Melbourne and Royal Children’s Hospital, Melbourne, Australia.

**Keywords:** continuous quality improvement, TB, community, PDSA framework

## Abstract

**SETTING:**

Daru Island in Papua New Guinea (PNG) has a high prevalence of TB and multidrug-resistant TB (MDR-TB).

**OBJECTIVE:**

To evaluate the early implementation of a community-wide project to detect and treat TB disease and infection, outline the decision-making processes, and change the model of care.

**DESIGN:**

A continuous quality improvement (CQI) initiative used a plan-do-study-act (PDSA) framework for prospective implementation. Care cascades were analysed for case detection, treatment, and TB preventive treatment (TPT) initiation.

**RESULTS:**

Of 3,263 people screened for TB between June and December 2023, 13.7% (447/3,263) screened positive (CAD4TB or symptoms), 77.9% (348/447) had Xpert Ultra testing, 6.9% (24/348) were diagnosed with TB and all initiated treatment. For 5–34-year-olds without active TB (*n* = 1,928), 82.0% (1,581/1,928) had tuberculin skin testing (TST), 96.1% (1,519/1,581) had TST read, 23.0% (350/1,519) were TST-positive, 95.4% (334/350) were TPT eligible, and 78.7% (263/334) initiated TPT. Three PDSA review cycles informed adjustments to the model of care, including CAD4TB threshold and TPT criteria. Key challenges identified were meeting screening targets, sputum unavailability from asymptomatic individuals with high CAD4TB scores, and consumable stock-outs.

**CONCLUSION:**

CQI improved project implementation by increasing the detection of TB disease and infection and accelerating the pace of screening needed to achieve timely community-wide coverage.

## Abstract (fr)

**CONTEXTE:**

L'île de Daru en Papouasie-Nouvelle-Guinée (PNG) présente une forte prévalence de la TB et de la TB multirésistante (MDR-TB).

**OBJECTIF:**

Évaluer la mise en œuvre précoce d'un projet à l'échelle de la communauté pour détecter et traiter la TB et l'infection, décrire les processus de prise de décision et changer le modèle de soins.

**CONCEPTION:**

Une initiative d'amélioration continue de la qualité (CQI, pour l’anglais « *continuous quality improvement* ») a utilisé un cadre de planification, d'action, d'étude, d'action (PDSA, pour l’anglais «*plan-do-study-act* ») pour la mise en œuvre prospective. Les cascades de soins ont été analysées pour la détection des cas, le traitement et l'initiation du traitement préventif de la TB.

**RÉSULTATS:**

Sur 3 263 personnes dépistées pour la TB entre juin et décembre 2023, 13,7% (447/3 263) ont été dépistées positives (CAD4TB ou symptômes), 77,9% (348/447) ont subi un test Xpert Ultra, 6,9% (24/348) ont reçu un diagnostic de TB et toutes ont commencé un traitement. Chez les 5 à 34 ans sans TB active (*n* = 1 928), 82,0% (1 581/1 928) ont subi un test cutané à la tuberculine (TCT), 96,1% (1 519/1 581) ont eu un test de dépistage du TCT, 23,0% (350/1 519) étaient positifs au TCT, 95,4% (334/350) étaient éligibles au TPT et 78,7% (263/334) ont initié le TPT. Trois cycles d'examen PDSA ont permis d'ajuster le modèle de soins, y compris le seuil CAD4TB et les critères TPT. Les principaux défis identifiés étaient l'atteinte des objectifs de dépistage, l'indisponibilité des expectorations chez les personnes asymptomatiques avec des scores CAD4TB élevés et les ruptures de stock de consommables.

**CONCLUSION:**

L'ACQ a amélioré la mise en œuvre du projet en augmentant la détection de la TB et de l'infection et en accélérant le rythme de dépistage nécessaire pour atteindre une couverture à l'échelle de la communauté en temps opportun.

TB remains a global public health crisis, with an estimated 1.3 million TB-related deaths in 2022.^1^ Papua New Guinea (PNG) is a low-middle-income country classified as a high-burden country for TB and multidrug/rifampicin-resistant TB (MDR/RR-TB) by the World Health Organization (WHO). In 2014, an unprecedented outbreak of MDR/RR-TB in Daru, South Fly District, Western Province, triggered a coordinated emergency response led by the PNG National Department of Health and Western Provincial Health Authority (WPHA).^2,3^ The TB case notification rate for South Fly District in 2022 was 693/100,000, and TB was hyperendemic in Daru (2,170/100,000) (WPHA reports). The proportion of MDR/RR-TB in South Fly District is approximately 20% of all TB diagnoses, higher than other settings in PNG.^3–5^

In 2015, a community-based model of care for TB detection and treatment, including MDR/RR-TB, was introduced in Daru, improving treatment outcomes and stabilising case notifications.^3^ Key interventions included community engagement, WHO-recommended rapid diagnostics (Xpert MTB/RIF and Xpert Ultra), new MDR-TB treatment regimens, a person-centred model of care, and enhanced data for decision-making. In 2017, screening and management of household contacts of TB cases were introduced, including TB preventive treatment (TPT) for well young children (<5 years) contacts of people with drug-susceptible (DS) TB. In 2019, TPT was introduced for child contacts of people with MDR/RR-TB.^6^ Despite progress, community transmission of TB in Daru, including MDR/RR-TB, continues,^3,7^ necessitating interventions to reduce transmission.

In 2023, following disruptions to TB services due to the COVID-19 pandemic, the WPHA and partners commenced a comprehensive community-wide strategy, the Systematic Island-Wide Engagement & Elimination Project for TB (SWEEP-TB) or Yumi Bung Wantaim Na Rausim TB Long Daru (Let’s Work Together to End TB in Daru.^8^ Under an operational research framework, this public health intervention aims to reduce TB incidence in Daru by combining the detection and treatment of TB disease and infection, including MDR-TB. The assessment will use routine surveillance and notification data comparing the age-related burden of TB and MDR-TB between the 24-month pre-intervention and the 24-month post-intervention period.

High screening, treatment, and TPT uptake and treatment completion rates are critical for effectiveness. We aimed to evaluate the early implementation of SWEEP-TB Daru using a CQI initiative. A PDSA framework and TB case detection and prevention cascades were utilised^9,10^ to monitor progress, identify gaps, and adjust the model of care.^11–14^

## METHODS

### Study design

This prospective implementation study has two components: a CQI initiative using a PDSA framework and a care cascade evaluation of TB case detection, treatment, and TPT uptake for SWEEP-TB Daru participants. The study was conducted from May to December 2023 on Daru Island in the South Fly District of the Western Province of PNG.

### Study setting and population

Daru is an island with an estimated population of 19,397^⋀^15. Residents live in overcrowded conditions with a crude population density of 2,500 persons/km^2^ and an average household size of 7.5.^15^ The WPHA leads the TB programme in Western Province with support from the National TB Program (NTP) and partners. Daru General Hospital is the only facility in the South and Middle Fly Districts that provides diagnosis and treatment services for MDR-TB. It has a 40-bed inpatient unit and one ambulatory TB clinic. Four community-based treatment centres at each ward on the island are staffed by nurses, community health workers, treatment supporters, and peer counsellors to provide community-based TB care.^16^ The study population was all Daru residents planning to live on the island for at least 12 months from household enumeration.

### Study procedures

The SWEEP-TB team conducted community-based engagement, screening, diagnostic, and prevention activities, data collection and management, and associated procurement and logistics. This team was integrated into the routine TB program, which provides treatment and care for active TB and household contact investigation and management. Routine systems were used for TB drug supply, laboratory diagnostics, and surveillance, as described elsewhere.^3^

A Community Advisory Group (CAG), self-named TB Nanito Kopia Kodu (the Voice to Kill TB Forever) with members representing key stakeholder groups: churches, schools, businesses, and key populations, provided community input and co-design for TB public health activities, including developing community education materials and advising on optimising acceptability, coverage, and inclusion of the intervention.^8^ Before implementation, an extensive community engagement and education campaign was conducted to improve foundational knowledge of TB transmission, infection, and disease and to explain the screening and prevention activities in SWEEP-TB. The study had an inclusion framework for people with disabilities, pregnant women, and children.

Household mapping and enumeration were conducted in the pre-implementation phase to geolocate households and elicit household details, including the number of people to be screened. Further information and education, including details of the model, were provided.

Screening was conducted in wards, the smallest administrative unit of government. In each ward, permission and support of ward leaders were obtained. A mobile TB case-finding clinic was deployed at multiple sites in each ward and included stations for education, screening, digital chest X-ray (CXR), clinical assessment, TPT, and vaccination. An innovative model of care was implemented using symptom screening, mobile digital CXR with computer-aided detection for TB (CAD4TB v6, Delft Imaging Systems, Netherlands), Xpert MTB/RIF Ultra (GeneXpert, Cepheid, Sunnyvale, USA), tuberculin skin testing (TST), and TPT for both DS and MDR/RR-TB strains using a novel combination regimen of six months of daily isoniazid and levofloxacin (6HLfx).^16^ Participants who screened positive underwent clinical evaluation at the SWEEP site and, if required, were referred to the diagnostic clinic at the hospital for diagnosis, including testing with Xpert Ultra. People diagnosed with active TB were started on treatment. Young child (<5 years) contacts without TB were offered TPT based on the susceptibility pattern of the index case as per the routine TB program. Persons aged 5–34 years with infection (TST positive) but not disease were offered a TPT regimen of 6HLfx. Those without evidence of TB disease or infection were offered Bacille Calmette-Guérin vaccination (BCG) if not previously vaccinated.

Person-centred TPT care was provided. Education and counselling were delivered by trained peers (TB survivors).^16^ Participants on TPT were provided medication for self-administration and reviewed monthly in clinics. An active drug safety and monitoring system was established for the novel TPT regimen. Participants were reimbursed K20 (∼USD 5) for participating in screening and K60 (∼USD 15) for completing a TPT course. Standard operating procedures for the model of care were developed and adjusted throughout implementation. Verbal consent was obtained for screening. Written consent was obtained for participants eligible for TST and TPT. Consent was sought from guardians of children under 18 years, with assent from children aged 8–17 years.

Four CQI cycles based on a PDSA framework were conducted during the study period. These involved regular review meetings with real-time reporting of TB cases and prevention cascades to monitor progress, followed by a facilitated discussion and feedback from the implementation team to identify strengths and bottlenecks and adjust the implementation of the model of care accordingly.

### Data collection and analysis

Data used for TB cascades were collected on paper-based forms and entered into an electronic medical records system (EMRS, Bahmni v.0.86; ThoughtWorks, Chicago, IL, USA).

Data were extracted from the EMRS system and analysed using R version 4.1 (R Foundation for Statistical Computing; Vienna, Austria). Demographic and clinical characteristics of people screened and diagnosed with active TB disease and TB infection were described as categorical variables by frequency and proportion.

TB case detection cascades during the study period were calculated using proportions based on numbers: 1) of participants who were screened; 2) who screened positive (symptoms, CAD4TB score >40, severe acute malnutrition); 3) who had diagnostic evaluation (sputum for Xpert Ultra assay); 4) diagnosed with active disease, and 5) initiated on treatment. TB prevention cascades reported on numbers: 1) of participants who were Daru residents aged 5–34 years without active TB (screened negative or who had active TB excluded after screening positive); 2) who had TST administered; 3) of TST read; 4) of TST positive; 5) eligible for TPT; and 6) initiated TPT. The proportion for each step was based on the denominator from the previous step and expressed as a percentage.

A standard template captured key discussions, actions, and changes to the model of care during PDSA cycle review meetings. Operational and implementation issues are documented in an issues tracker (Microsoft Excel) in real time using thematic categories—screening and diagnosis, treatment, and prevention. The PDSA cycle was described according to the four cycles. Changes to the model of care were described using the thematic categories.

### Ethics approval

Ethical approval was obtained from the PNG Medical Research Advisory Committee (MRAC 22.04) and the Alfred Hospital Ethics Committee (568/22).

## RESULTS

**FIGURE 1.**
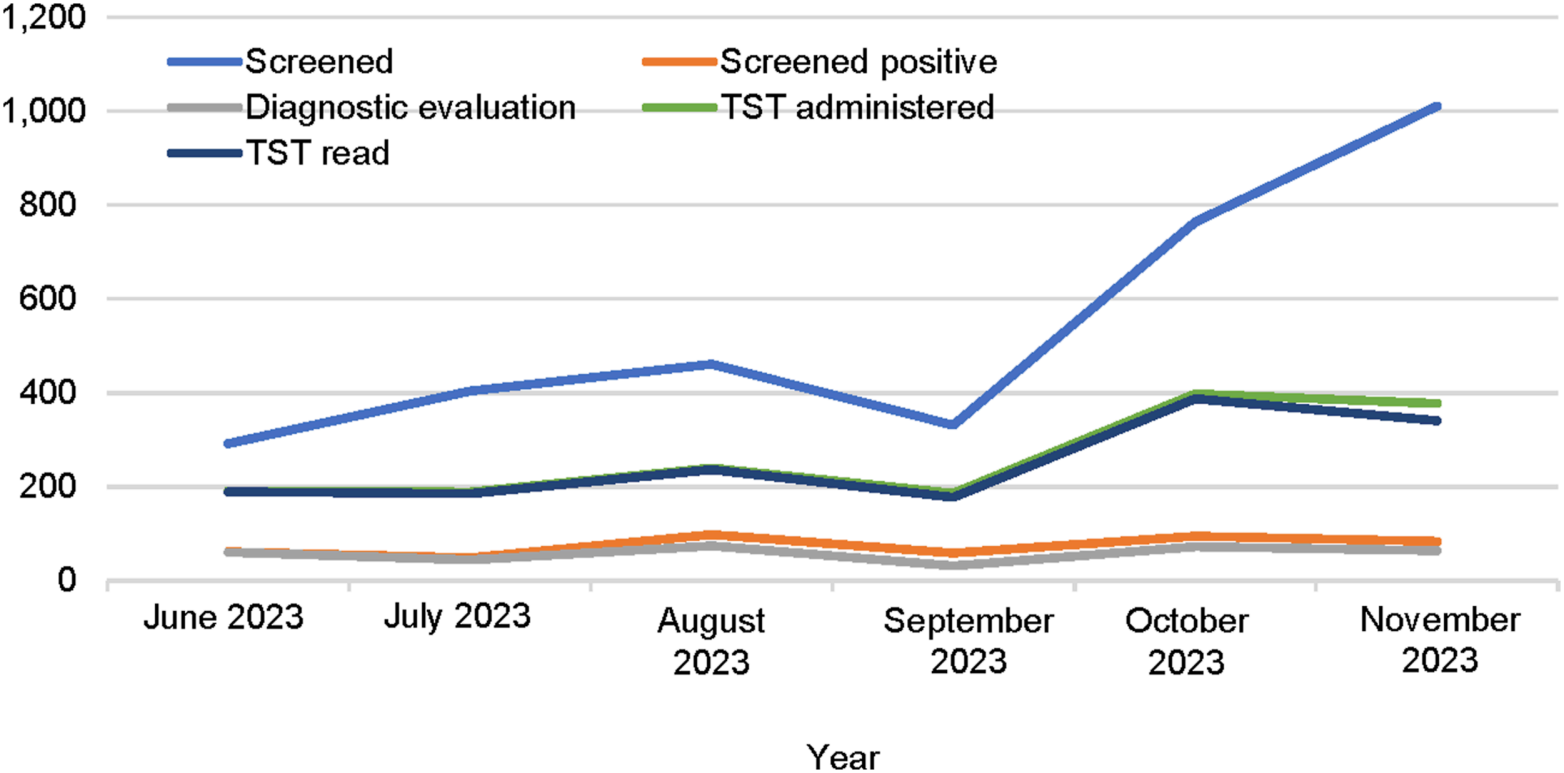
Implementation of SWEEP-TB Daru activities by month, June–November 2023. SWEEP-TB = Systematic Island-Wide Engagement & Elimination Project for TB; TST = tuberculin skin test.

In Daru, 3,263 community participants were screened for TB from June to November 2023. The number screened represents 14.8% of the 22,099 people enumerated during island-wide household mapping. Participant characteristics for 3,159 with complete data (Table 1) were 51% female, 58% aged 5–34 years, and 97% had received BCG. A history of TB contact was reported by 13%, and previous TB treatment and TPT were reported by 9% and 1%, respectively. The number of participants screened per month increased from 292 in June to 1,011 in November (Figure 1).

**TABLE 1.** Characteristics of participants screened in the community during SWEEP-TB Daru, June to November 2023.

Characteristic	(*n* = 3,159)^*^
	*n* (%)
Sex	
Female	1,607 (51)
Male	1,552 (49)
Age group, years	
0–4	402 (13)
5–14	825 (26)
15–24	522 (17)
25–34	474 (15)
35–54	665 (21)
55+	266 (8)
Unknown	5 (0.2)
Nutritional assessment	
Normal	1,850 (59)
Moderate	290 (9.2)
Severe	10 (1.0)
Overweight	219 (6.9)
Unknown	734 (23)
BCG vaccination	
Yes	3,080 (97)
No	16 (1)
Unknown	63 (2)
History of TB treatment	
Yes	268 (9)
No	2,844 (90)
Unknown	47 (1)
History of TPT	
Yes	37 (1)
No	3,037 (96)
Unknown	85 (3)
Contact with known case	
Yes	418 (13)
No	2,741 (87)
Reported one or more symptoms	
Yes	328 (10)
No	2,831 (90)

* Excludes 104 participants with incomplete data entry at the time.

SWEEP-TB = Systematic Island-Wide Engagement & Elimination Project for TB; BCG = bacille Calmette-Guerin; TPT = TB preventive therapy.

**FIGURE 2.**
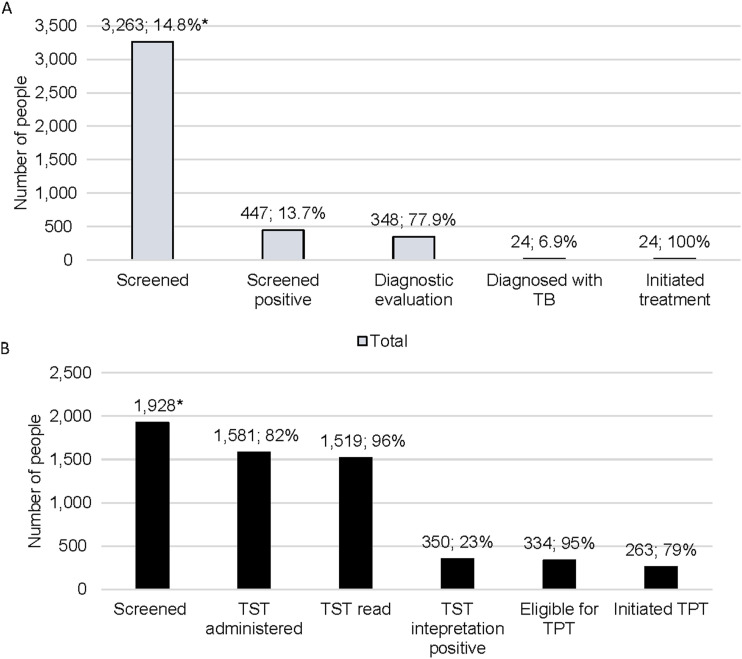
SWEEP-TB Daru case detection and prevention cascades, June–November 2023. **A)** TB case detection cascade, June-November 2023. Screening yield: 24/3,263 (0.7%); diagnostic yield: 24/447 (5.4%). *Proportion of 22,099 people enumerated during household mapping. The denominator for each proportion is the total from the previous step of the cascade; **B)** TB prevention cascade, June–November 2023. *The number screened is of those aged 5–34 years and active TB ruled out. The denominator for each proportion is the total from the previous step of the cascade. TST = tuberculin skin test; TPT = tuberculosis preventive therapy.

The TB case detection cascade is presented in Figure 2A. Of 3,263 participants, 13.7% (447/3,263) screened positive by symptoms and/or CAD4TB score. The majority (77.9%) underwent further evaluation for TB and provided a sputum sample for Xpert Ultra. Twenty-four TB cases were detected, 23 bacteriologically confirmed and one clinically diagnosed, representing a screening yield of 0.7% and a diagnostic yield of 5.4%. All detected cases were linked to treatment.

There were 1,928 participants eligible for a TST (aged 5–34 and screened negative or screened positive but did not have TB diagnosed after further evaluation). The number of TST placements and reading increased over time, peaking in October 2023 (Figure 1). The overall proportion of TST reading was high at 96.1%, and uptake of TPT was high at 78.7% (Figure 2B).

Four PDSA cycles were completed before and during implementation (baseline, Month 1–2; Month 3–5; Month 6) (Table 2). Cycle 0 (baseline) shows the initial model review during the pre-implementation phase. The focus was on reviewing tools, standard operating procedures, training materials, and human resources. Cycle 1 reflects further changes in the revised model of care. Key challenges identified during the Cycle 1 review include a slow screening pace, diagnosis and management of asymptomatic participants with a high CAD4TB score who did not have bacteriological confirmation, and supply chain management leading to stockouts of Xpert Ultra cartridges, TPT drugs, PPD, and other consumables. Recommendations and changes led to Cycle 2 and 3 reviews with a plan for better efficiencies in the next implementation phase.

**TABLE 2.** PDSA cycle in SWEEP-TB Daru implementation, May–December 2023.

Cycle	Plan	Do	Study	Act
0: Pre-implementation (May 2023)	Trial implementation	Pilot implementation conducted: November 2022	Use of unique identifiers (ID, image-based) for participants trialled but not considered user-friendly by participants or for health information system Trialled screening with Daru Provincial Hospital (DPH) staff households but low uptake	Switch to a more user-friendly numerical SWEEP ID for participants. Planned for regular SWEEP education and awareness sessions and updates for healthcare workers at DGH
	Test tools and process in trial implementation	Version 1 of tools and SOP used in trial implementation	Used previous household mapping but was outdated Reviewed tools and SOP Need to reduce prolonged visits and improve screening flow	Full household mapping planned. Updated SOP and tools Updated database for data capturing (Bahmni) Revised and documented screening flow in SOP
	Team recruitment, training, and orientation	33 new staff recruited, total SWEEP-TB staff (*n* = 45); SWEEP-TB staff trained and orientated	Staff assessed after training and orientation for sub-team allocation as per organogram	Allocated in sub-teams to focus on specific activities/tasks
	Staff screening in preparation for full implementation in the community	57 staff screened in May 2023	Revised process and tools with team	Refresher training on specific areas for improved practice Prepare logistics of community implementation
1: Start implementation (1 June–22 August 2023)	Full implementation by ward: sequence: Ward 1 > Ward 2 > Ward 3 > Ward 4	Ward 1: Community engagement resumed on 10 May 2023 Household mapping commenced on 10 May 2023 Community screening commenced on 13 June 2023	Mismatch, 3 screening teams vs. 1 clinical team Slow screening pace, projected to not reach targets Discrepancies in Screening & Diagnostic Form identified Data entry to the EMRS lagging – being done at the office and not field Stock out of drugs (isoniazid 100 mg) and consumables (Xpert Ultra cartridges) PPD cold-chain breach Security threat: team verbally assaulted/ threatened during community awareness and mapping activities	Reassess staffing requirements to increase clinical teams - TB fellows recruited and planned for additional HEO and CHWs Monitoring screening reports of the number screened and refused per team. Increased screening frequency from 3 days/week to 5 days/week Explore options for increasing data entry pace: field data entry through a VPN connection; explore hiring additional DEOs, and bi-weekly data cleaning reports for data and screening/clinical teams Close monitoring of drugs and consumables stock through the hospital and consider a backup plan/parallel system for drugs and consumables CAG/community leaders/community reps to be involved in the initial week of screening at a new location to encourage community participation and mitigate security risks
2: Implementation (23 August–7 November 2023)	Progress implementation with recommended actions from Cycle 1	Continued screening and TPT in Ward 1	CAG provided feedback on community education and engagement materials, including consent Main challenges were with CXR and CAD4TB connections, PPD stockouts, and duplicate screening Challenges with the referral pathway and links with TBDC SWEEP-TB staffing complement still not optimum, impacting operations at the screening station Data entry still lagging with data quality concerns Staff wellbeing at community/screening site concerns	Verbal consent for screening is to be obtained from the household head. Increase age of consent from 16 to 18 years. Human resources: TB Fellows x2 commenced and rotated to support SWEEP-TB 2x CHW replacements recruited to fill vacancies from resignations Mapping team absorbed into screening/clinical team to increase HR capacity Additional data entry officer recruited Data: Weekly follow-up day (Thursday) to complete pending screening activities and pending data entries in the tracker Monthly data cleaning day to complete quality checks and data entries/updates in EMRS CXR: Observe the systematic approach of switching on/off CXR and CAD4TB UPS for backup power Repair van CXR machine Expedite purchase of ultraportable CXR machine Staff wellbeing: Trailer for storage and mobilisation of current mobile CXR machine with generator set Bicycles for participant follow-ups in the community Temporary screening and TPT shelters to supplement gazebos Staff toilet, wash and break station
3: Implementation (8 November–15 December 2023)	Continue and progress implementation with recommended actions from Cycle 2	Progressed screening in Ward 1	Main challenges included TPT drug (levofloxacin) shortage resulting in treatment interruptions and increased backlog of pending initiations, and screening delays or disruptions due to numerous factors, e.g. lack of communication or coordination across other teams Persisting challenges were lag in data entries, prolonged waiting time at screening stations as the number screened increased, delays in screening site preparations Other challenges included duplicate screening, increase in the number of participants pending screening outcome, incorrect nutritional assessment, gaps in routine TPT referral, and concerns for confidentiality	Escalated issue of levofloxacin shortage for decisions on the way forward for both supply and patient care. Raised issue of poor communication or coordination with respective teams concerned to prevent/mitigate delays or disruptions in screening Strengthen weekly follow-up and monthly data cleaning days to clear backlogs of screening outcome pending and EMRS data entry Plan for refresher training on gaps identified or areas to improve, meanwhile conduct on-the-job training/refreshers for nutritional assessment, routine TPT referrals, and maintaining confidentiality of participants Identified options to mitigate duplicate screening

PDSA = plan-do-study-act; SWEEP-TB = Systematic Island-Wide Engagement & Elimination Project for TB; DGH = Daru General Hospital SOP = standard operating procedure; EMRS = Electronic Medical Record System; HEO = Health Extension Officer; CHW = Community Health Worker; VPN = virtual private network; DEO = Data Entry Officer PPD = purified protein derivative; CAG = Community Advisory Group; UPS = uninterruptible power supply; CXR = chest X-ray; CAD4TB = computer-aided diagnostics for TB; TBDC = TB Diagnostic Centre; TPT = TB preventive therapy.

The changes in the model of care for the community-wide screening, treatment, and prevention of TB are described in Table 3. Most notable changes are 1) modified consent procedures to engage the household head and increase the age of consent from 16 to 18 years based on CAG advice, 2) the CAD4TB threshold reduced from 50 to 40 as a baseline before an assessment can be made for an ideal threshold for the project, 3) exclusion of participants from TPT eligibility who were previously treated for TB or took TPT within the last 12 months, and 4) use of a shield in pregnancy during CXR procedures and avoiding TPT in the first and second trimesters of pregnancy (Table 3).

**TABLE 3.** Changes in the SWEEP-TB Daru model of care over time during a continuous quality improvement initiative.

Category	Activity	Initial	Pre-implementation	Month 1: Implementation	Month 2: Implementation	Month 3: Implementation
Screening and diagnosis	Consenting (screening)	Obtain consent before screening. Age of consent = 16 years Group TB education and information session and individual consenting/assent	Written consent in the household Age of consent = 18 years Group TB education and individual information sessions and consenting	Verbal consent was obtained in the household Age of consent = 18 years Group TB education and individual information session and consent/assent Assessment for capacity to consent included	No change	No change
	Symptom screening	Symptom screening at household	No change	No change	No change	No change
	CXR (CAD4TB)	CXR for all participants CAD4TB for ≥10 years CAD4TB score threshold 50	CXR for all participants CAD4TB for ≥10 years CAD4TB score threshold 50	CXR for ≥10 years CXR for <5 if presumptive (symptom positive, SAM), or contact of index patient (DS/DR) CAD4TB for ≥10 years CAD4TB score threshold 40	No change Pregnant participants undergo CXR after the second trimester CAD4TB low, but CXR image abnormal; referred to hospital for further evaluation CAD4TB high but unable to produce sputum, referred to hospital for further evaluation	Order protective shield for pregnant women
	Sputum collection (Xpert Ultra)	Sputum collection at screening site Sputum collection for all participants	Same as initial GA/FNAB referred to hospital for sample collection	No change Sputum collection only for presumptive participants	No change Paediatric referrals for GA, FNAB weekly	No change
	Clinical evaluation	Clinical assessment Review of results (CXR, Xpert Ultra) Assign outcome (TB/no TB) Refer to hospital for active TB treatment initiation	Same as initial Screening result (presumptive/not presumptive)	No change Referrals to hospital for further evaluation (presumptive but unable to produce sputum, recently completed active TB treatment with abnormal CXR)	No change	No change Include severe acute malnutrition (SAM) as presumptive
	Consenting (TST/TPT)	Written consent at the screening site	Same as initial	No change Group information session Individual capacity to consent	Exclusion if previous TB treatment or TPT in the past 12 months	No change
	TST	Eligible: 5–34 years old, <5 contacts of DR-TB index patients, permanent residents of Daru or residing in the next 12 months TST placement after ruling out active TB TST reading within 48–72 h	Same as initial	TST placement before screening outcome, on the first day of screening Others same as initial	TST placement for all participants aged 5–34 years TST reading for missed at 72 h, done up to day 7 from placement Others no change	No change Strengthen referral from routine contact tracing for <5 contacts of DR-TB index patients Strengthen referral to routine TPT for <5 contacts of DS-TB index patients
Treatment	MTB/RR-TB detected	Refer to hospital for active TB treatment	Same as the initial follow-up to confirm treatment initiation	Same as pre-implementation	Same as pre-implementation	Same as pre-implementation
	Presumptive, MTB not detected	Based on clinical evaluation	Refer to hospital for further evaluation	Same as pre-implementation	Same as pre-implementation	Same as pre-implementation
Prevention	TPT	TST-positive initiated on 6HLFx (5–34 years) and 6Lfx (<5 years)	TPT initiation done at the screening site Follow-up clinics at DART sites (community treatment centres)	Follow-up clinics at the screening site	TPT considerations for pregnant participants	Follow-up clinics at DART site Alternative regimen proposed for 6HLfx during TPT stockout was 6H
	BCG	0–34 years who have never been vaccinated	No change	No change	No change	No change

CXR = chest radiography; CAD4TB = computer-aided diagnostics for TB; SAM = severe acute malnutrition; DS-TB = drug-susceptible TB; DR-TB = drug-resistant TB; GA = gastric aspirate; FNAB = fine-needle aspirate biopsy; TST = tuberculin skin test; TPT = TB preventive therapy; MTB = *Mycobacterium tuberculosis*; RR-TB = rifampicin-resistant TB; 6HLfx = 6 months of isoniazid and levofloxacin; DART = Daru Accelerated Response to TB.

## DISCUSSION

Early experiences from implementing SWEEP-TB Daru, a comprehensive community-wide initiative to detect, treat, and prevent TB, have demonstrated an effective model with high uptake rates. Notably, there have been reasonable rates of linkage to evaluation for active disease and TB infection among those eligible, complete linkage of diagnosed cases to active TB treatment, and high rates of TPT uptake among eligible (TST-positive) children, adolescents, and adults. The yield of active TB and TB infection among community participants screened is moderately high, supporting the continuation of the initiative. Although our study has only completed four cycles by the time of this interim analysis, findings and lessons from the CQI initiative optimised the implementation of SWEEP-TB. However, there is room for improvement. High uptake and completion across all stages of the detection and prevention cascades will be required to reduce TB incidence. We have found the PDSA cycle to be an effective framework for CQI that will be continued as part of SWEEP-TB Daru.

Community-wide, systematic household screening, treatment, and prevention of TB is novel for PNG and the region. Implementation in a population with such a high prevalence of MDR/RR-TB is also novel, with specific challenges such as overcrowding and a highly mobile population.^8^ As the context and scope of this community-wide project are innovative and unique and only partially implemented, it is difficult to compare to the final findings from other programmes. A community-wide intervention in a remote Pacific island with a smaller population had a similar screening yield (0.75%) and introduced TPT for household contacts with infection.^17^ Across the border in a district of Indonesia’s Papua province, a CQI approach was employed to successfully strengthen and decentralise TB services with increased case detection at the primary care level and the introduction of TPT, but this was also limited to eligible household contacts in a setting with a low prevalence of MDR/RR-TB.^18^ Similar comprehensive search, treat, and prevent initiatives^19^ are now being implemented in the Asia-Pacific region but not in high transmission MDR/RR-TB settings.^20,21^

In Daru, the pace of screening coverage needs to be accelerated further to achieve impact. This has been a major focus of the implementation team and the CQI initiative. A protracted intervention is likely to be less effective at reducing transmission and burden than a rapid one, but by how much is not known. Annual screening performed for three years in communities in southern Vietnam significantly impacted TB prevalence and transmission measured at four years after intervention compared to non-intervention communities.^22^ The resources required for multiple, rapid active case-finding (ACF) cycles are considerable and well beyond the usual capacity of TB programmes. The impact on TB notifications over time from this “real-world” implementation project in PNG and similar community-wide approaches^20,21^ in the region will be informative.

A model of care was developed for SWEEP-TB with CAG and broad stakeholder input before implementation, and adjustments were made during implementation. We found that most changes to the model of care were required for the screening component during the four phases of revision. This may be because a greater proportion of issues were identified in activities relating to screening compared to treatment and prevention. In addition, this analysis reports the initial phase of implementation with fewer issues identified for treatment and prevention activities.

We found that around 20% of participants were lost to care in the case detection (from screen positive to diagnostic evaluation) and prevention (TPT eligible to initiated) cascades. In our setting, “structural barriers” such as social and financial issues and population mobility have been identified as barriers to accessing TB services rather than inadequate knowledge or health-seeking behaviours.^23^ All people diagnosed with TB were linked to the routine TB program for treatment registration and continuation of care, and those receiving TPT are being routinely and regularly followed to completion. However, this interim analysis is too early to report on treatment outcomes, including infection with TPT.

We identified high BCG coverage for the population screened thus far. Infant BCG immunisation has been recommended in PNG for decades but with low uptake in the past few years.^24^ In our study, evidence of BCG coverage included examination for a typical scar rather than relying only on recall. Most participants in SWEEP-TB are adolescents and adults, so our findings reflect mainly past rather than recent immunisation coverage or uptake. Nonetheless, the high coverage is an encouraging finding given that low childhood immunisation coverage in PNG and Western Province is a major public health concern.^24^

A strength of this study is the use of systematically recorded data for real-time evaluation, monitoring, and evaluation. Data-informed change was used to monitor the impact of the change. The high yield from case detection and prompt initiation of treatment indicates the quality of care provided. The willingness of individuals to participate and overall acceptance of SWEEP-TB by the community may reflect the sustained and recent efforts to engage and educate the community over many years, including input from the CAG and involvement of community leaders, likely leading to a sense of community “ownership” and less TB-related stigma.^2,3,8^ One limitation is a potential bias in our reporting, as the primary focus of the CQI approach was towards improving outcomes with positive results. However, identified challenges and negative outcomes or data did inform the process as barriers to overcome. Early analysis of implementation data may not be representative of the final analysis. However, for the CQI framework, continuous monitoring of cascade data is necessary and important to guide implementation. There are no data yet for the outcomes of TB treatment or TPT. These key programmatic indicators will be analysed to report full cascades of care. In conclusion, implementing the CQI initiative using a PDSA framework improved project implementation. Detection of TB disease and infection in island residents increased, and the pace of screening was needed to achieve timely community-wide coverage.
